# Constructing nitrided interfaces for stabilizing Li metal electrodes in liquid electrolytes

**DOI:** 10.1039/d1sc01806j

**Published:** 2021-06-01

**Authors:** Zhijie Wang, Yanyan Wang, Chao Wu, Wei Kong Pang, Jianfeng Mao, Zaiping Guo

**Affiliations:** Institute for Superconducting & Electronic Materials, Australian Institute for Innovative Materials, University of Wollongong NSW 2522 Australia; School of Chemical Engineering and Advanced Materials, The University of Adelaide Adelaide South Australia 5005 Australia zaiping.guo@adelaide.edu.au

## Abstract

Traditional Li ion batteries based on intercalation-type anodes have been approaching their theoretical limitations in energy density. Replacing the traditional anode with metallic Li has been regarded as the ultimate strategy to develop next-generation high-energy-density Li batteries. Unfortunately, the practical application of Li metal batteries has been hindered by Li dendrite growth, unstable Li/electrolyte interfaces, and Li pulverization during battery cycling. Interfacial modification can effectively solve these challenges and nitrided interfaces stand out among other functional layers because of their impressive effects on regulating Li^+^ flux distribution, facilitating Li^+^ diffusion through the solid-electrolyte interphase, and passivating the active surface of Li metal electrodes. Although various designs for nitrided interfaces have been put forward in the last few years, there is no paper that specialized in reviewing these advances and discussing prospects. In consideration of this, we make a systematic summary and give our comments based on our understanding. In addition, a comprehensive perspective on the future development of nitrided interfaces and rational Li/electrolyte interface design for Li metal electrodes is included.

## Abstract 2

In this perspective, we make a systematic summary and give out our comments on constructing nitrided interfaces for stabilizing Li metal electrodes.

## Graphical abstract

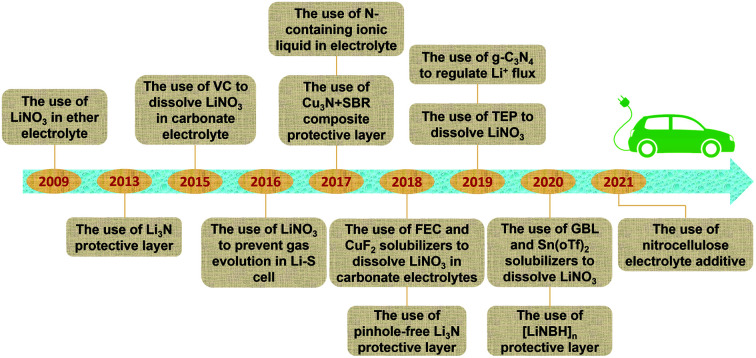

## 1. Introduction

**Fig. 1:**
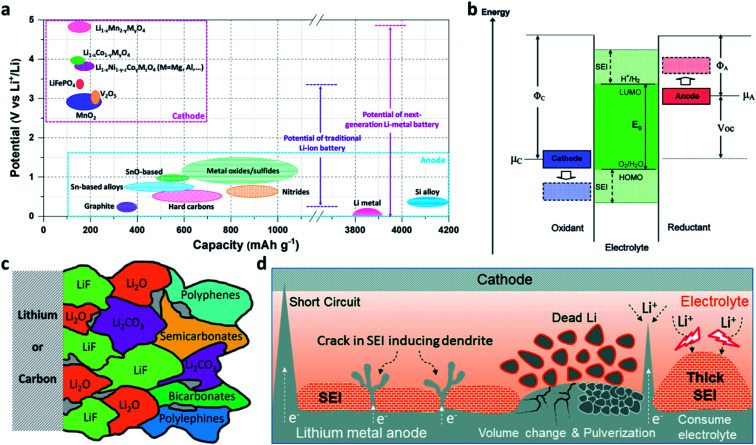
Opportunities and challenges of Li metal electrodes. (a) Voltage *versus* capacity for anode and cathode materials of Li-ion batteries and Li–metal batteries; (b) schematic of the open-circuit energy diagram of the electrolyte window (*E*_g_) and the chemical potentials *μ*_A_ and *μ*_C_ of the anode and cathode; *μ*_A_ > LUMO and/or *μ*_C_ < HOMO, where the HOMO is the highest occupied molecular orbital.^3^ Reproduced with permission. Copyright 2008, American Chemical Society. (c) Illustration of the SEI formed on Li or graphite.^30^ Reproduced with permission. Copyright 2021, John Wiley and Sons. (d) Schematic illustration of the formation of Li dendrites, damage to the native SEI, and the formation of porous electrochemically non-active Li.^38^ Reproduced with permission. Copyright 2020, Elsevier.

Rechargeable lithium (Li) ion batteries (LIBs) have been shaping many aspects of our modern life.^1–4^ Nevertheless, the traditional graphite-based LIBs have nearly reached their theoretical limit in energy density (∼250 W h kg^−1^), which hinders the development of portable electrical devices and electric vehicles.^5–8^ Li metal has the lowest electrochemical potential (−3.04 V *vs.* the standard hydrogen electrode (SHE)) among the alkali metals and a much higher theoretical specific capacity of 3860 mA h g^−1^ (which is 10 times that of graphite) (Fig. 1a).^4,9–15^ When paired with high-voltage cathode materials, Li metal batteries (LMBs) are able to provide a 5 V-class output voltage and a 500 W h kg^−1^-class energy density (Fig. 1a).^16–19^ Therefore, reviving LMBs is an effective strategy to break the performance limitation of LIBs.^20–23^ The main challenge is that all liquid electrolytes are thermodynamically unstable at 0 V *vs.* Li/Li^+^, because the lowest unoccupied molecular orbital (LUMO) of the electrolyte is lower than the Fermi level of Li metal (Fig. 1b).^3,24^ Thus, the electrolyte accepts electrons from Li metal and reductively decomposes on the surface of the Li electrode to form a solid-electrolyte interphase (SEI).^16,25–28^ The inner layer of the SEI (close to Li metal) consists of inorganic components such as lithium oxide (Li_2_O), lithium fluoride (LiF), and lithium carbonate (Li_2_CO_3_), while the outer layer of the SEI (close to the electrolyte) mainly consists of organic components such as polyolefins and semicarbonates (Fig. 1c).^25,29,30^ The SEI layer is electrically non-conductive but ionically conductive, so that it can block the electron transport at the Li/electrolyte interface and stop the further decomposition of the electrolyte while Li^+^ diffuses through the layer.^16,26^

Unlike graphite which stores Li^+^ in its lattice with acceptable volumetric changes (∼12%), the Li metal anode accommodates Li^+^ at the Li/electrolyte interface, leading to unlimited volumetric changes during Li plating/stripping processes.^11,12,31,32^ Unfortunately, the native SEI formed on Li is brittle, so it fails to tolerate the stress caused by the volumetric changes of Li metal electrodes.^25,33–35^ In addition, Li^+^ is preferentially deposited on the protuberant tips with stronger electrical fields on the substrate, leading to the formation and growth of dendritic Li.^26,36^ An Li dendrite has a high Young's modulus of ∼5 GPa,^37^ so it can easily pierce the SEI. Once the SEI is damaged, the newly exposed Li would immediately react with the electrolyte to form a new SEI.^16^ Meanwhile, the cracked SEI layer may also expose defects and in turn accelerate the deposition of Li on the defects and form new Li dendrites. Furthermore, Li stripping from the roots of the dendrite would break the electrical contact and produce porous “dead” Li.^38^ With battery cycling and continuous SEI build-up, the above problems lead to electrolyte depletion, loss of electrochemically active Li, Li electrode pulverization, and battery performance decay (Fig. 1d).^38,39^ Even more troubling, the Li dendrites could lead to internal short-circuits or even safety hazards in working batteries (Fig. 1d).^26,38,40–42^

Interfacial engineering is critical to stabilize Li metal electrodes.^16,20,43^ Constructing nitrided interfaces on the surface of Li electrodes or current collectors has been proved to be effective to suppress Li dendrite formation and growth, as well as protecting Li from electrolyte erosion. The nitrided interfaces can regulate Li^+^ flux distribution near the Li electrodes or current collectors, facilitate Li^+^ diffusion through the SEI, and passivate the reductive surface of Li, thus improving the electrochemical performance of LMBs. In this paper, we review our current fundamental understanding and recent advances in developing nitrided interphases for stabilizing Li metal electrodes. The strategies for constructing nitrided interphases, including building artificial SEI layers, electrolyte engineering, substrate modification, and separator functionalization, have been comprehensively summarized and discussed. In addition, our perspective on the future development of nitrided interfaces and rational Li/electrolyte interface design for Li metal electrode is included.

## 2. Advantages of nitrided interfaces

### 2.1. Functions of nitrided interfaces

#### 2.1.1. Regulating the Li^+^ flux distribution

Practical Li metal electrodes or current collectors are rough and uneven. The protuberant tips on the substrate (Li or current collector) have stronger electrical fields. Li^+^ is preferentially deposited on these protuberant tips, leading to the formation and growth of dendritic Li.^12,36^ Regulating the uniform deposition of Li^+^ is an important step to eliminate the safety risks and performance decay caused by Li dendrites. Nitrogen (N) has lone-pair electrons and can act as a Lewis base site to adsorb positively charged Li^+^ (Lewis acidic site), thus creating a lithiophilic surface on the Li metal electrode and decreasing the over-potential for Li plating. In addition, N has a high electronegativity (*χ*) of 3.04, and when bonded with atoms with lower electronegativity, such as boron (B) (*χ* = 2.04) and carbon (C) (*χ* = 2.55), the electron cloud in N–C or N–B polar covalent bonds will migrate to the N side. The increased charge density around N can further improve the interaction between N and Li^+^. Therefore, the nitrided interface is able to regulate the Li^+^ flux distribution near the Li electrode surface or current collectors and thus guide Li^+^ uniform deposition.

#### 2.1.2. Facilitating Li^+^ diffusion through the SEI

In general, Li^+^ is solvated with four to six solvent molecules in the electrolyte.^44,45^ Before plating onto the substrate (the Li anode or current collectors), the solvated Li^+^ is firstly de-solvated near the SEI, and then the naked Li^+^ ions migrate across the SEI.^45–47^ The migration speed is the rate-determining step in the Li deposition process.^48^ The high Li^+^ ionic conductivity of the SEI helps to improve the kinetics of the Li plating process and thus helps to enhance the electrochemical performance of Li metal electrodes. The diffusion mechanism of Li^+^ through the SEI is complicated and controversial. It was proved that LiF, Li_2_O, and Li_2_CO_3_ in the native SEI diffuse Li^+^*via* grain boundaries, as their intrinsic ionic conductivity is relatively low (up to ∼10^−9^ S cm^−1^).^25,49–51^ Nitrides such as lithium nitride (Li_3_N) and LiN_*x*_O_*y*_ have much higher ionic conductivity (up to ∼10^−3^ S cm^−1^),^52–54^ and they can provide faster Li^+^ migration channels in the SEI. Therefore, the nitrided SEI can facilitate Li^+^ diffusion and improve the kinetics of the Li plating process.

#### 2.1.3. Passivating the active surface of Li metal electrodes

As mentioned, the formation of the SEI blocks the electron tunneling at the Li/electrolyte interface and thus stops the decomposition of the electrolyte. The thickness of the SEI is related to its electrical conductivity. Nitrides such as Li_3_N, LiN_*x*_O_*y*_, carbon nitrides (C_3_N_4_), and nitrided polymers all have an ultralow electrical conductivity.^54^ When used to modified the surface of the Li metal electrode, they can physically and electrically isolated Li from the electrolyte and thus passivate the reductive surface of the Li metal electrode, which helps to reduce the thickness of the SEI.

### 2.2. Comparison of nitride interfaces with other strategies

Besides nitride interfaces, metal oxides (such as MgO),^55^ phosphates (such as Li_3_PO_4_),^56,57^ some lithium halides (such as LiCl and LiI),^56,58,59^ and lithium chalcogenides (such as Li_2_S and Li_2_Se)^60,61^ have also been introduced as modification layers on Li metal. Generally, their precursors are hardly soluble in non-aqueous electrolytes, and as a consequence, these layers are usually fabricated by *ex situ* methods, serving as artificial SEI layers. These modification layers do play a positive role in protecting the Li metal, but they may be damaged by the interfacial stress during Li plating/stripping processes and therefore lose their functions. In contrast, in the case of nitride interfaces, some of their precursors (such as nitrates, amides, N-containing ionic liquids, and nitrocellulose) are soluble in certain non-aqueous solvents, which enables continuous repair of nitride interfaces during battery cycling when these precursors are introduced into the electrolyte as solvents or additives.

Fluorinated interfaces, which feature LiF-rich SEI layers, are widely acclaimed for their outstanding effects on Li metal protection, which is based on their high Young's modulus and the high interfacial energy of LiF.^49,62^ Although fluorinated interfaces are excellent for inhibiting side reactions between Li metal and the electrolyte, nitride interfaces still show advantages over them in some aspects, especially in bulk ionic conductivity. The transport of Li ions in LiF is much more difficult than in Li_3_N or LiN_*x*_O_*y*_, obviously limiting the grain growth of deposited Li during the plating process. According to the morphologies, deposited Li with a nitrided interface has a larger grain size but smaller microstructural tortuosity compared with Li with a fluorinated interface, contributing to higher reversibility of the active Li during battery cycling.

## 3. Constructing a nitrided artificial SEI on Li metal electrodes

### 3.1. Methods to construct a nitrided SEI on Li metal electrodes

Since the formation of the SEI is a key factor in controlling the surface properties of Li, one of the effective approaches to stabilize the Li metal electrode is to construct functional artificial SEI layers on its surfaces.^28,35,43,63,64^ According to the preparation mechanism, the strategies to develop a nitrided artificial SEI can be divided into physical methods and chemical methods.

#### 3.1.1. Physical methods

**Fig. 2:**
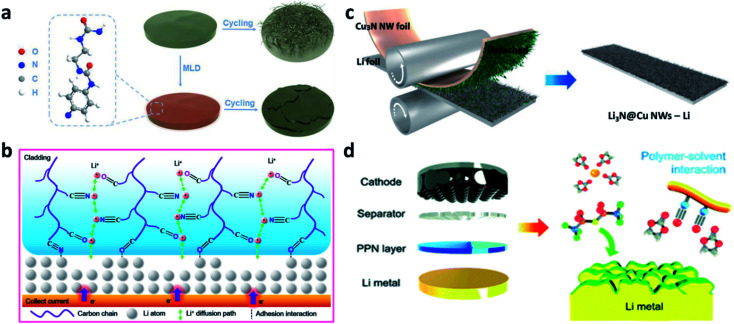
Constructing artificial SEI layers on Li metal electrodes *via* physical methods. (a) Schematic illustration of “polyurea” deposited on Li to guide Li uniform deposition;^65^ reproduced with permission. Copyright 2019, John Wiley and Sons. (b) Illustration of P(BMA-AN-St) cladding regulating Li^+^ flux;^66^ reproduced with permission. Copyright 2019, American Chemical Society. (c) Fabrication of a Cu_3_N layer on Li foil *via* physical rolling and printing method;^67^ reproduced with permission. Copyright 2020, John Wiley and Sons. (d) Schematic illustration of coating the PPN layer on Li metal in the battery assembly process.^68^ Reproduced with permission. Copyright 2019, Royal Society of Chemistry.

Physical pre-coating methods, such as doctor blading, physical pressing, drop coating, atomic layer deposition (ALD), *etc.*, are simple approaches to easily prepare nitrided interfaces on Li metal electrodes. For instance, a polyurea thin layer was coated on Li metal *via* the ALD method.^65^ The abundant N-containing polar groups in the polyurea were believed to be able to redistribute the Li^+^ flux and lead to a uniform plating/stripping process (Fig. 2a). A poly(butylmethacrylate-acrylonitrile-styrene) (P(BMA-AN-St)) cladding was drop coated on the Li surface.^66^ Benefiting from the affinity of the polar groups (C≡N and C=O) in the polymer chains with both Li^+^ and Li metal, the P(BMA-AN-St) cladding provided channels for regulating the Li^+^ (Fig. 2b),^66^ so that a dendrite-free surface and improved electrochemical performance of Li metal electrodes were realized, even with deep cycling. Paik *et al.* modified copper nitride nanowires (Cu_3_N NWs) on Li foil through one-step roll pressing. The Cu_3_N NWs could be conformally printed onto the Li metal and form a Li_3_N@Cu NW layer on the Li electrode (Fig. 2c).^67^ Yu *et al.* synthesized a polar polymer network (PPN) layer and coated it on a Li metal electrode during battery assembly.^68^ The C≡N groups of polyacrylonitrile polymer chains in the PPN could reduce the high reactivity of the C=O groups of carbonate solvents and promote the decomposition of salt anions (PF_6_^−^ and bis(trifluoromethane)sulfonimide (TFSI^−^)), forming a stable SEI (Fig. 2d). In addition to these artificial SEI layers, other different inorganic artificial SEIs were also prepared *via* physical methods on Li metal electrodes. For instance, a Li_3_N layer can be coated on the Li metal electrode *via* pressing and rubbing Li_3_N powder to suppress Li dendrite formation.^69^ Yang *et al.* coated a layer of acid-treated graphitic (g)-C_3_N_4_ on Li, and its N-containing groups were able to rearrange the concentration of Li^+^ and enhance the transfer of Li^+^.^70^

It should be pointed out that the thickness of the nitrided artificial SEI developed *via* physical methods is normally more than a few micrometres (as summarized in Table 1), which would certainly impose a sacrifice on the overall volumetric energy density of Li metal electrodes. In addition, the physical methods could not well control the homogeneity of the artificial SEI on Li metal electrodes, and the adhesion between the SEI and the Li metal would not be strong enough, which may lead to the exfoliation of the artificial SEI during battery cycling. Besides, the organic artificial SEI layers modified by physical methods have poor ionic conductivity, so they normally lead to high electrochemical polarization for Li metal electrodes.

**Table 1:** Summary of nitrided artificial SEIs for Li metal electrodes and the symmetric cell performance

Artificial SEI	Thickness	Fabrication method	Electrolyte	Current density (mA cm^−2^)	Capacity (mA h cm^−2^)	Lifespan (h)	Polarization (mV)	Ref.
Polar polymer network	N/A	Physical pressing in battery assembly	LiTFSI : EC = 1 : 10	10	1	200	∼300	68
Polyurea	∼4 nm	Atomic layer deposition	1 M LiPF_6_ in EC/DEC/DMC	1	2	400	∼170	65
P(BMA-AN-St)	∼4 μm	Drop coating	1 M LiPF_6_ in EC/DEC/DMC	0.5	1	800	∼200	66
Acid-treated g-C_3_N_4_	∼5 μm	Physical pressing	1 M LiTFSI in DOL/DME with 2 wt% LiNO_3_	1	1	400	∼240	70
Li_3_N	N/A	Pressing and rubbing	1 M LiPF_6_ in EC/DEC	1	2	360	∼240	69
Cu_3_N nanowires	∼3 μm	Roll-printing	1.3 M LiPF_6_ in EC/DEC with 5% FEC	3	1	250	∼240	67
AgNO_3_	N/A	Drop coating	1 M LiTFSI in DOL/DME with 1 wt% LiNO_3_	5	0.5	50	∼400	83
PEO–UPy	70 nm	Drop coating	1 M LiTFSI in DOL/DME with 2 wt% LiNO_3_	5	10	1000	300	80
CTF + LiI	∼20 μm	Drop coating	1 M LiPF_6_ in EC/DEC	10	1	500	500	77
Li_3_N	N/A	N_2_ flow treatment	1 M LiPF_6_ in EC/DMC	N/A	N/A	N/A	N/A	71
Li_3_N	8.25 μm	N_2_ flow treatment	1 M LiPF_6_ in EC/DMC	N/A	N/A	N/A	N/A	72
Pinhole-free Li_3_N	50–400 nm	N_2_ based reaction	1 M LiTFSI in DOL/DME with 1 wt% LiNO_3_	N/A	N/A	N/A	N/A	74
Li_3_N	∼8 μm	Plasma activation under N_2_	1 M LiPF_6_ in EC/DMC	0.5	1	500	∼250	73
LiPON	250 nm	N_2_ plasma-assisted deposition	1 M LiTFSI in DOL/DME with 1 wt% LiNO_3_	3	1	600	∼160	75
N-organic@Li_3_N	950 nm	C_3_N_4_ based surface reaction	1 M LiTFSI in DOL/DME with 1 wt% LiNO_3_	1	2	1100	∼80	78
PECA–Li_3_N/LiN_*x*_O_*y*_	∼4 μm	*In situ* polymerization of ECA with a LiNO_3_ additive	1 M LiPF_6_ and EC/DMC	1	1	200	∼160	79
[LiNBH]_*n*_	140–160 nm	Two-step dehydrogenation reaction	1 M LiTFSI in DOL/DME with 1 wt% LiNO_3_	3	1	700	204	82

#### 3.1.2. Chemical methods

**Fig. 3:**
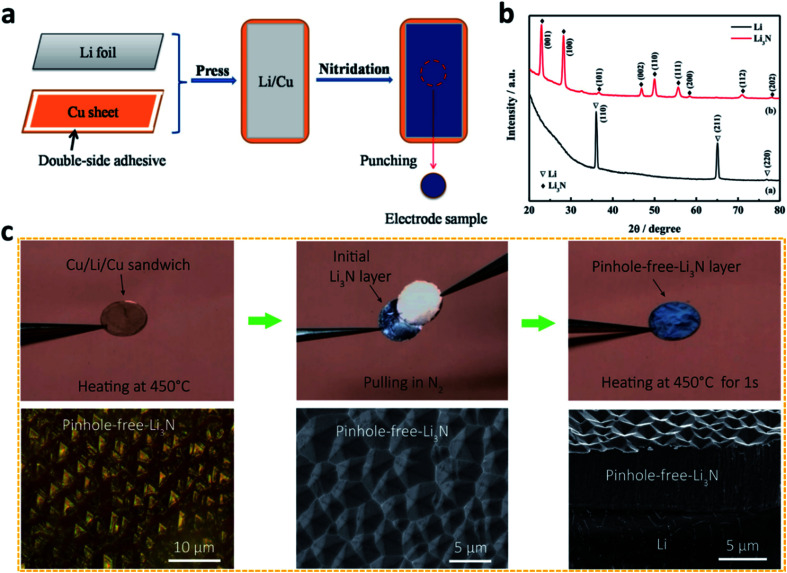
Constructing artificial SEI layers on Li metal electrodes *via* chemical methods. (a) Preparation of a Li_3_N film on an Li surface by utilizing the reaction between N_2_ gas and Li metal; (b) characterization of the Li_3_N film by X-ray diffraction (XRD);^72^ reproduced with permission. Copyright 2015, Elsevier. (c) Preparation of a pinhole-free Li_3_N layer to protect the Li metal electrode, and optical and scanning electron microscope (SEM) images of the pinhole-free Li_3_N layer.^74^ Reproduced with permission. Copyright 2018, American Chemical Society.

By using chemical reactions between Li and N-containing precursors, more dense and homogeneous artificial SEI layers can be prepared. The most common nitrided artificial SEI developed by a chemical method is Li_3_N. The first reported chemical method to develop a Li_3_N layer was using a N_2_ gas flow to treat Li in a desiccator.^71^ It was proved that an electrochemically stable Li_3_N protective layer had been coated on Li metal by this method. Furthermore, Tu *et al.* heated Li chips in a tube furnace under a N_2_ flow (Fig. 3a), and the formation of Li_3_N on Li metal was confirmed from the X-ray diffraction patterns (Fig. 3b).^72^ They revealed that the Li_3_N layer could efficiently prevent contact between Li and the electrolyte and reduce the side reactions. Similarly, Zhou *et al.* grew a highly [001] oriented, flower-like Li_3_N film on Li metal by an N_2_ plasma activation method.^73^ Because of its high Young's modulus and high ionic conductivity, the Li_3_N film can physically block direct contact between the reactive Li metal and the liquid organic electrolyte.

Despite these achievements, the effect of Li_3_N is limited to some extent by its small grain size (<160 nm), which leads to weak interconnections between the Li_3_N particles. To solve this challenge, Cui *et al.* heated Li metal in a N_2_ atmosphere at a high temperature to develop a pinhole-free Li_3_N layer on the Li metal surface (Fig. 3c).^74^ The dense, large, and strongly interconnected grains of Li_3_N in the film reduced the defects in the artificial SEI and effectively improved the stability of the Li metal electrode during battery cycling.

Apart from forming Li_3_N, Xie *et al.* also dropped AgNO_3_/tetrahydrofuran (THF) solution on the Li surface, and the AgNO_3_ particles would further react with Li to form LiNO_3_, which is useful for regulating Li^+^ plating behaviour and suppressing Li dendrite growth. A lithium phosphorus oxynitride layer on a Li metal anode with high ionic conductivity and chemical stability was developed *via* a nitrogen plasma-assisted deposition method to suppress the corrosion from the electrolyte and promote uniform Li plating/stripping.^75^

As summarized in Table 1, the nitrided artificial SEI layers prepared *via* chemical methods are generally inorganic, and most of them are thinner as well as having higher ionic conductivity, so they could reduce the polarization of Li metal electrodes. These inorganic artificial SEI layers are normally brittle, however, so the integrity of the SEI would be damaged by the interfacial stress changes caused by Li plating/stripping processes, which would shorten the lifespan of Li metal electrodes.

### 3.2. Building nitrided organic–inorganic composite artificial SEIs

**Fig. 4:**
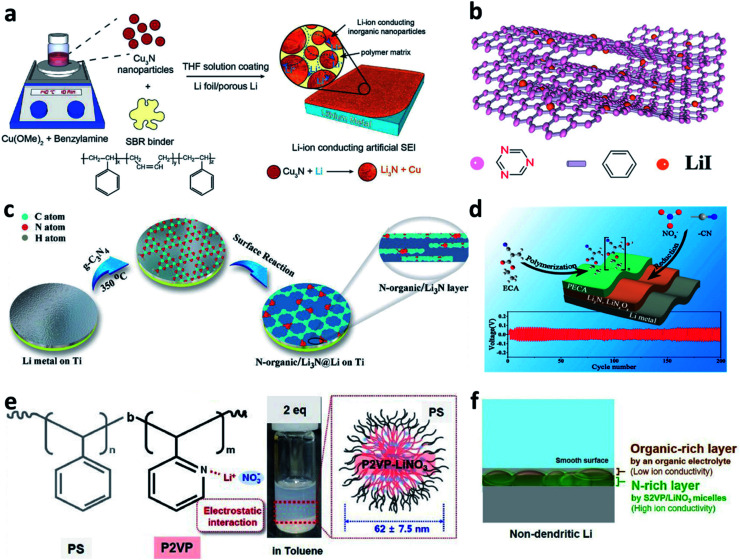
Constructing nitrided organic–inorganic composite interfaces for Li metal electrodes. (a) Preparation of a Cu_3_N-SBR interface on a Li metal electrode;^76^ reproduced with permission. Copyright 2017, John Wiley and Sons. (b) Structural illustration of the CTF–LiI composite layer;^77^ reproduced with permission. Copyright 2020, John Wiley and Sons. (c) Preparation of an N-organic/Li_3_N composite layer on a Li metal electrode;^78^ reproduced with permission. Copyright 2020, John Wiley and Sons. (d) Schematic illustration of the preparation of a PECA–Li_3_N/LiN_*x*_O_*y*_ dual protection layer for a Li metal electrode and the subsequent electrochemical performance.^79^ Reproduced with permission. Copyright 2017, American Chemical Society. (e) Chemical structure, optical image, and schematic illustration of the S_2_VP/LiNO_3_ micelles; (f) schematic illustration of the multilayered structure formed on the surface of S_2_VP/LiNO_3_–Li.^81^ Reproduced with permission. Copyright 2021, Elsevier.

Building nitrided organic–inorganic composite interfaces is a good idea that takes advantage of the merits of both individual components and overcome their disadvantages. In this regard, Cui *et al.* developed a reactive interface constructed from Cu_3_N nanoparticles joined together by styrene butadiene rubber (SBR) as an artificial SEI for Li metal electrodes.^76^ The inorganic Cu_3_N has high ionic conductivity, and the organic SBR has high mechanical strength and high flexibility (Fig. 4a). The Cu_3_N further reacted with Li and a composite artificial SEI composed of Li_3_N/SBR/Cu was formed on the surface of the Li metal electrode. The Li_3_N particles provided ionically conductive paths, while the SBR confined the Li_3_N particles and buffered the volume changes of the Li anode. Zheng *et al.* coated a covalent triazine framework (CTF)-LiI hybrid artificial SEI on Li by the doctor blade method (Fig. 4b).^77^ The N in CTF could bind with Li^+^ from the electrolyte to form Li–N bonds and thus facilitate uniform Li deposition. The uniformly distributed LiI particles could help to improve the mechanical stress to suppress Li dendrite growth. Yu *et al.* reported a composite artificial SEI consisting of an N-containing organic phase (N-organic) and an inorganic Li_3_N phase by utilizing the hyperthermal reduction of Li and g-C_3_N_4_ (Fig. 4c).^78^ The obtained N-organic phase could link with the Li_3_N phase and form a conformal and compact coating on Li. The authors believed that the C–N=C and N–(C)_3_ groups realized the homogeneous distribution of Li^+^ and provided nucleation sites for Li deposition, while the Li_3_N reduced the resistance to Li^+^ transfer across the Li/electrolyte interfaces. Besides, a dual-layer artificial SEI was constructed *via in situ* polymerization of ethyl α-cyanoacrylate (ECA) monomers on the Li metal surface, in which LiNO_3_ was introduced with the ECA monomers as an additive (Fig. 4d).^79^ The CN^−^ groups in ECA and the LiNO_3_ additive reacted with Li to form a nitrided inorganic interface on Li during battery cycling. Poly(ethyl α-cyanoacrylate) (PECA) was used to cover the outer surface to accommodate the volume changes and buffer the interfacial stress during the Li plating/stripping processes. Xiong *et al.* modified a self-healing supramolecular copolymer, which consisted of pendant poly(ethylene oxide) (PEO) segments and ureido-pyrimidinone (UPy) quadruple-hydrogen-bonding moieties, on a Li metal electrode *via* a drop coating method.^80^ During the following drying process, the amide and heterocyclic amine groups in PEO–UPy polymer reacted with Li metal and formed a stable artificial SEI (LiPEO–UPy) layer on the Li metal electrode. The developed LiPEO–UPy layer could protect the electrolyte from side reactions and homogenize the fast Li^+^ flux to the surface of the Li metal. Lee *et al.* developed hybrid polyion complex micelles and coated them on Li foil, in which ionized LiNO_3_ combined with block copolymer micelles, polystyrene-*block*-poly(2-vinyl pyridine) (S2VP), *via* electrostatic interaction (Fig. 4e).^81^ It was believed that the S2VP polymer could isolate the active Li from carbonates so as to reduce the side reaction between them, and meanwhile, the introduced LiNO_3_ could further dissolve into the electrolyte during battery cycling. As a result, a composite N-rich SEI with a multilayered structure could be formed on the Li electrode (Fig. 4f). With the designed protective layer, Li metal full cells with a high voltage cathode (LiNi_0.8_Co_0.1_Mn_0.1_O_2_) delivered superior performance, even under harsh test conditions (thin Li anode, high areal-capacity of 4.0 mA h cm^−2^, and high current density of 4.0 mA cm^−2^).

**Fig. 5:**
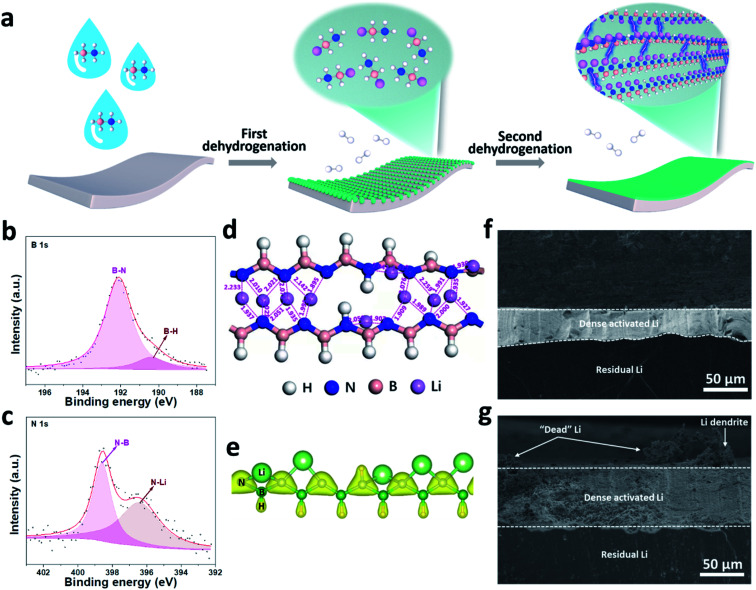
Building a [LiNBH]_*n*_ artificial SEI for a Li metal electrode. (a) Schematic illustration of the fabrication process for the [LiNBH]_*n*_ layer; surface XPS spectra of B 1s (b) and N 1s (c) for the [LiNBH]_*n*_ coated Li; (d) relaxed atomic configurations of two adjacent [LiNBH]_*n*_ chains with bond lengths between Li and N atoms (the unit of the values is Å), which reveals that the Li–N distances are comparable to the corresponding bond lengths in the crystalline phases of Li_3_N; (e) charge density distribution in the chain of [LiNBH]_*n*_ with an isosurface of 0.15 e Å^−3^, which confirms that the N is negatively charged; cross-sectional SEM images of (f) bare Li and (g) Li@[LiNBH]_*n*_ after 20 cycles.^82^ Reproduced with permission. Copyright 2020, John Wiley and Sons.

Despite the advantages of organic/inorganic composite artificial SEIs, it is challenging to control the homogeneous distribution of organic and inorganic phases. To overcome this, our group synthesized a multi-functional [LiNBH]_*n*_ layer as an artificial SEI for Li metal anodes by utilizing a two-step dehydrogenation reaction between Li and ammonia borane (Fig. 5a–c), which features the properties of both organic and inorganic SEIs.^82^ The obtained ASEI is composed of [LiNBH]_*n*_ chains, which are cross-linked and self-reinforced by their intermolecular Li–N ionic bonds, and thus give rise to a flexible nature (Fig. 5d). Because of the higher charge density of N in the polar [LiNBH]_*n*_ chain, Li^+^ from the electrolyte will be absorbed by the N to form additional Li–N ionic bonds, which helps to regulate the homogeneous distribution of the Li^+^ flux on Li electrodes (Fig. 5e). In addition, the [LiNBH]_*n*_ layer is electrically isolated but has high ionic conductivity, thus facilitating Li^+^ diffusion and deposition beneath the artificial SEI layer. Therefore, with the protection of the [LiNBH]_*n*_ layer, Li dendrite growth has been successfully suppressed and a denser and flatter surface was achieved after Li plating/stripping cycles (Fig. 5f and g).

In a short summary, inorganic nitrided artificial SEI layers have high ionic conductivity and relatively low thickness, but they suffer from low integrity and mechanical flexibility. Organic nitrided artificial SEI layers (normally prepared *via* physical methods) can regulate the Li^+^ flux distribution and buffer the volume changes of Li metal electrodes, while their poor ionic conductivity and high thickness usually lead to high electrochemical polarization. Building nitrided inorganic–organic composite artificial SEI layers is useful to take advantage of the individual components. Most of the composite artificial SEI layers delivered enhanced lifespan compared with pure organic or pure inorganic artificial SEIs. Unfortunately, it is still difficult to control the homogeneous distribution of organic and inorganic components in the SEI. In addition, the thickness of artificial SEI layers varies from a few nanometres to tens of micrometres. To avoid the sacrifice of the volumetric energy density, the thickness of the artificial SEI should be lower than one micrometre, especially considering that the anodes in practical LMBs are thin foils with a thickness of <50 μm. Another big challenge to artificial SEI layers is their stability during battery cycling. With continuous Li plating/stripping cycles, the structure and the integrity of artificial SEI layers would be destroyed by the interfacial mechanical strength, particularly during long-term cycling. Therefore, improving the stability, reducing the thickness, and increasing the flexibility are critical to boost the practical applications of nitrided artificial SEI layers in LMBs.

## 4. Electrolyte engineering

The deposition behaviour of Li^+^ is strongly related to the physical and chemical properties of the electrolyte.^16,45,48^ Electrolyte engineering, including introducing functional additives, using new solvents, regulating the Li^+^ solvation structure, *etc.*, is useful to stabilize Li metal electrodes. The most efficient method to evaluate the effects of these electrolytes is to test Li plating/stripping reversibility in Li–Cu cells.

### 4.1. LiNO_3_ additive

**Fig. 6:**
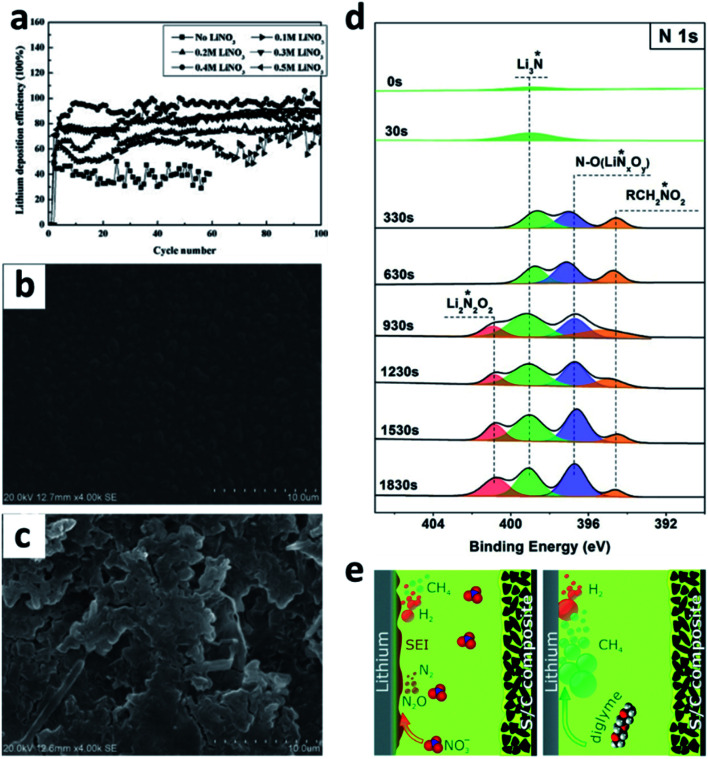
The use of a LiNO_3_ additive in ether-based electrolytes. (a) The effects of the LiNO_3_ additive towards improving the CE of Li–Cu cells in the ether electrolyte; comparison of SEM images of the Li electrode cycled in the ether electrolyte with LiNO_3_ (b) and without LiNO_3_ (c);^84^ reproduced with permission. Copyright 2011, Elsevier. (d) Surface characterization by XPS of the Li electrode cycled in the ether-based electrolyte with the LiNO_3_ additive;^85^ reproduced with permission. Copyright 2014, Elsevier. (e) Schematic illustration of the passivation of the Li surface and the suppression of gas evolution in the ether electrolyte by the LiNO_3_ additive.^86^ Reproduced with permission. Copyright 2016, Royal Society of Chemistry.

LiNO_3_ is a practical and economical additive to improve the electrochemical performance of LMBs. It was first used in an ether-based electrolyte to suppress the shuttle effect of polysulphides in Li–S batteries by Aurbach *et al.*^87^ They studied the surface of the Li anode cycled in the LiNO_3_-containing ether electrolyte and proposed that the LiNO_3_ additive was decomposed on Li to form LiN_*x*_O_*y*_ and LiOR. Wen *et al.* further proved that LiNO_3_ is able to improve the coulombic efficiency (CE) of the Li plating/stripping processes in Li–Cu cells (Fig. 6a), as well as suppressing Li dendrite growth.^84^ A smoother surface of the Li metal anode was obtained after adding 0.4 M LiNO_3_ into the ether-based electrolyte (Fig. 6b and c). By using the more accurate and sensitive X-ray photoelectron spectroscopy (XPS) depth profile method, Xiong *et al.* revealed that LiNO_3_ in the ether-based electrolyte is reduced on the Li metal surface and forms a complex product consisting of Li_3_N, LiN_*x*_O_*y*_, and RCH_2_NO_2_ (Fig. 6d).^85^ Brezesinski *et al.* also demonstrated that LiNO_3_ can form a protective layer on the Li metal anode and suppress gas evolution in the Li–S battery in conjunction with a diglyme-based electrolyte. In particular, the amount of flammable CH_4_ and H_2_ is dramatically decreased, and either very little or no H_2_ is generated during discharge (Fig. 6e).^86^

**Fig. 7:**
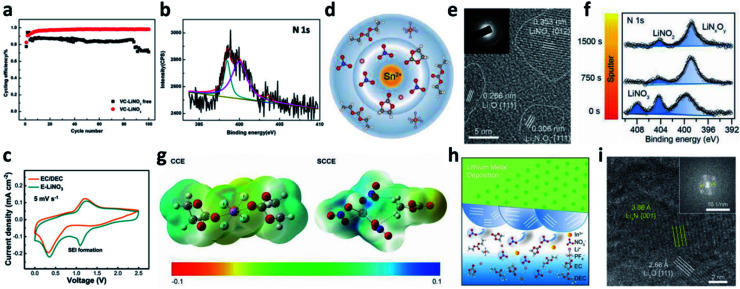
The use of the LiNO_3_ additive in carbonate-based electrolytes. (a) CE of a Li–Cu cell in a carbonate electrolyte with and without VC–LiNO_3_ as the additive and (b) N 1s XPS spectrum of the cycled Li;^90^ reproduced with permission. Copyright 2015, Elsevier. (c) Cyclic voltammetry (CV) curves of Li–Cu cells with and without CuF_2_–LiNO_3_ co-additives, where an additional peak belonging to LiNO_3_ decomposition at ∼1.1 V can be observed;^91^ reproduced with permission. Copyright 2018, John Wiley and Sons. (d) Structural illustration of the Sn^2+^ solvated sheath; (e) high resolution TEM (HRTEM) image of the SEI formed in the carbonate electrolyte with Sn(OTf)_2_–LiNO_3_ additives, with the corresponding selected area electron diffraction pattern in the inset; (f) N 1s XPS depth profiles for the SEI formed in the electrolyte with Sn(OTf)_2_–LiNO_3_ additives;^35^ reproduced with permission. Copyright 2020, John Wiley and Sons. (g) Electrostatic potential (ESP) images for the solvated EC and DEC molecules in the electrolyte with and without In(OTf)_3_–LiNO_3_ as an additive; (h) schematic illustration of the formation of an inorganic wavy SEI; (i) cryo-TEM image of the inorganic wavy SEI showing the presence of Li_3_N.^92^ Reproduced with permission. Copyright 2020, John Wiley and Sons.

Even though LiNO_3_ has achieved a big success in ether-based electrolytes and it also has good solubility in ether-based electrolytes (up to ∼5 wt%), its application in high-voltage carbonate-based electrolytes is limited due to its ultralow solubility in carbonate solvents (lower than 10^−5^ g mL^−1^).^88,89^ To boost the application of the LiNO_3_ additive in carbonate-based electrolytes, various solubilizers have been utilized to promote its dissolution in carbonate solvents. It was initially reported that 2% vinylene carbonate (VC) can promote the dissolution of 0.1 M LiNO_3_ in an ethylene carbonate/dimethyl carbonate (EC/DMC)-based electrolyte and effectively improve the reversibility of Li plating/stripping processes in Li–Cu cells (Fig. 7a).^90^ By analysing the surface of the cycled Li metal electrode with XPS, the existence of Li_3_N in the SEI was confirmed (Fig. 7b). Huang *et al.* further used a trace amount of CuF_2_ to promote the dissolution of 1 wt% LiNO_3_ in an EC/diethyl carbonate (DEC)-based electrolyte, and proved that LiNO_3_ was reduced on Li and formed a nitrided SEI (Fig. 7c).^91^

Increasing the concentration of LiNO_3_ in carbonate-based electrolytes could improve the electrochemical performance of LMBs. In this regard, Lu *et al.* used 0.5 wt% Sn(OTf)_2_, where OTf is trifluoromethanesulfonate, as a solubilizer to increase the solubility of LiNO_3_ in carbonate electrolytes to as high as 5 wt%.^35^ Tin(ii), which is a Lewis acid, can effectively coordinate NO_3_^−^ and promote complete dissociation between ion pairs without decomposing the solvent molecules (Fig. 7d). By using high-resolution transmission electron microscopy (TEM, Fig. 7e) and XPS depth profiling (Fig. 7f), they confirmed that N-containing species, such as Li_3_N and LiN_*x*_O_*y*_, were formed in the SEI of the Li metal electrode. With the benefits of the nitrided SEI, the CE in Li–Cu cells was improved to 98.14% at a high capacity of 3 mA h cm^−2^ over 150 cycles, and the cycling performance of Li‖NCM811 full cells delivered superior electrochemical performance under practical conditions. Similarly, they also used In(OTf)_3_ to dissolve 3 wt% LiNO_3_ in a carbonate-based electrolyte and achieved a high CE of >98% in Li–Cu cells at a high plating capacity of 4 mA h cm^−2^.^92^ They demonstrated that, because of the presence of In^3+^, the reactivity of the EC molecule was reduced, and the NO_3_^−^ anions were more likely to undergo a site-selective reaction at the inner Helmholtz plane and form an N and O-rich inorganic wavy SEI (Fig. 7g and h), which was experimentally proved by cryo-TEM results (Fig. 7i).

**Fig. 8:**
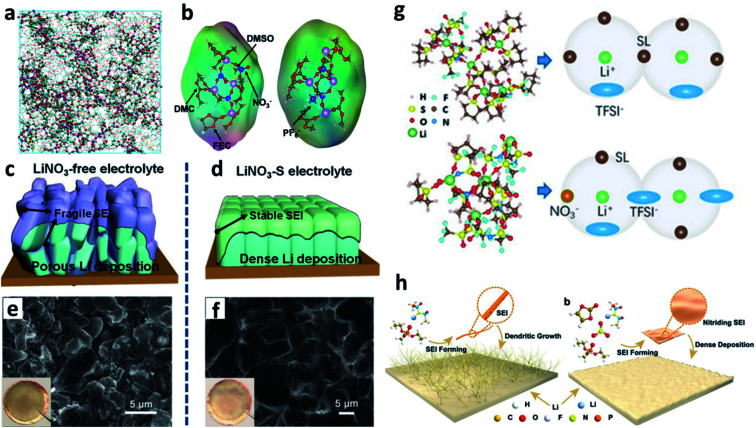
The use of the LiNO_3_ additive in other ester-based electrolytes. (a) MD simulation of a carbonate electrolyte with LiNO_3_/DMSO as the additive; (b) structure of the Li^+^ solvated sheath; schematic illustration of the SEI and Li deposition in the electrolyte without the LiNO_3_/DMSO additive (c) and with the LiNO_3_/DMSO additive (d); SEM images and corresponding optical images of deposited Li from the electrolyte without the LiNO_3_/DMSO additive (e) and with the LiNO_3_/DMSO additive (f);^93^ reproduced with permission. Copyright 2020, John Wiley and Sons. (g) MD simulations and the Li^+^ solvation sheath of a sulfolane-based electrolyte with and without LiNO_3_;^94^ reproduced with permission. Copyright 2020, John Wiley and Sons. (h) Schematic illustration of building a nitrided interface on a Li metal electrode by adding LiNO_3_ into a triethyl phosphate-based electrolyte.^95^ Reproduced with permission. Copyright 2019, John Wiley and Sons.

The use of extra solubilizers has improved the solubility of LiNO_3_ in a carbonate-based electrolyte, but they also increase the cost of the electrolyte. In addition, the solubilizers could be reduced on Li, so that they may destabilize the SEI. Sulfones (such as dimethyl sulfoxide (DMSO), sulfolane, *etc.*) have high solvability towards LiNO_3_, so they can replace the extra solubilizers and be used as solvents in the electrolyte to dissolve LiNO_3_. In this aspect, Wang *et al.* used DMSO solvent to dissolve LiNO_3_ and prepared a 4 M LiNO_3_/DMSO solution as an additive.^93^ They added 5 wt% of this additive into a carbonate-based electrolyte and achieved an ultrahigh CE of 99.55% in Li–Cu cells. It was indicated that distinct NO_3_^−^ anions were involved in the Li^+^ solvation sheath, and a small number of DMSO molecules were also found in the Li^+^ solvation sheath (Fig. 8a and b). The NO_3_^−^ in the solvation sheath could be reduced on the Li surface and formed a nitrided inorganic-rich SEI, which was more stable than the SEI formed in the LiNO_3_-free electrolyte (Fig. 8c and d), while the DMSO molecules could not be decomposed. Therefore, denser and more compact plated Li was obtained on the Cu substrate (Fig. 8e and f). Wang *et al.* also used pure sulfolane as the solvent in their electrolyte for LMBs, which contained 3.25 M lithium bis(trifluoromethanesulfonyl)imide (LiTFSI) as a salt and 0.1 M LiNO_3_ as an additive.^94^ By using molecular dynamics (MD) simulations, they pointed out that the NO_3_^−^ anions in the Li^+^ solvation sheath could promote the coordination of TFSI^−^ anions with Li^+^ (Fig. 8e). During battery cycling, these anions in the Li^+^ solvation sheath would be reduced and formed an inorganic SEI. It should be emphasized that LiNO_3_ is strongly oxidizing, so it will increase the safety risk of the battery after being added into the electrolyte, although most of the reported work failed to mention this safety issue. To address this problem, Guo *et al.* used triethyl phosphate as a solvent to dissolve 1 M LiNO_3_ into the electrolyte as well as an extinguishant to eliminate fire risk.^95^ The developed electrolyte not only generated a nitrided SEI that could suppress Li dendrite growth (Fig. 8f), but also improved the safety of the resultant LMBs. The CE for Li plating/stripping processes only reached ∼97%, however, which was not high enough for practical LMBs.

In short, the use of LiNO_3_ as an additive has effectively optimized the SEI and improved the Li plating/stripping reversibility. The application of LiNO_3_ in high-voltage and more practical carbonate-based electrolytes for LMBs is limited, however, due to its low solubility. Different solubilizers were used to increase the solubility of LiNO_3_ in carbonate-based electrolytes, although these solubilizers increase the cost of the electrolyte and their decomposition on Li would destabilize the SEI. LiNO_3_ has high solubility in organic phosphate esters, sulfones, and amides, and they can be used as solvents or liquid solubilizers to dissolve LiNO_3_ in the electrolyte. The thermodynamic stability of these solvents is poorer than that of carbonate solvents, however, which increases the undesirable side reactions between the electrolyte and the Li metal electrode. Furthermore, for the development of safe and practical LMBs, the fire risk caused by the oxidizing properties of LiNO_3_ should be carefully considered.

### 4.2. Other N-containing additives

**Fig. 9:**
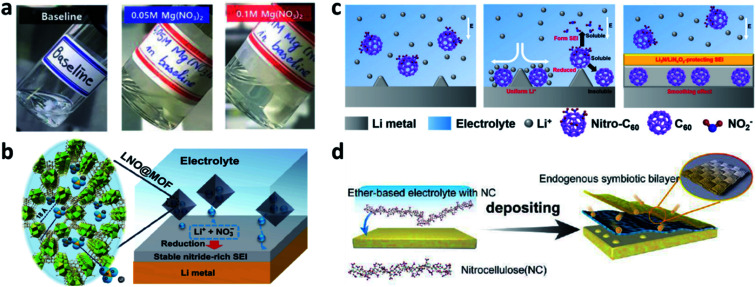
The use of other electrolyte additives for constructing nitrided interfaces on Li metal electrodes. (a) The use of Mg(NO_3_)_2_ as an additive in a carbonate electrolyte;^96^ reproduced with permission. Copyright 2020, John Wiley and Sons. (b) Schematic illustration of the use of MOF-808/LiNO_3_ as an electrolyte additive for LMBs;^97^ reproduced with permission. Copyright 2020, Springer Nature. (c) Schematic illustration of nitro-C_60_ as a bifunctional electrolyte additive for LMBs;^98^ reproduced with permission. Copyright 2019, American Chemical Society. (d) Illustration of the endogenous symbiotic Li_3_N/cellulose double SEI using nitrocellulose.^99^ Reproduced with permission. Copyright 2021, John Wiley and Sons.

Apart from LiNO_3_, some other N-containing additives can also be used to build a nitrided SEI on Li metal electrodes. In this regard, Sun *et al.* reported that Mg(NO_3_)_2_ can be dissolved in a carbonate-based electrolyte as an additive.^96^ They suggested that Mg(NO_3_)_2_ can be dissolved directly as Mg^2+^ and NO_3_^−^ ions in the electrolyte even at a concentration of 0.1 M, which was quite different from the situation for LiNO_3_ (Fig. 9a). The NO_3_^−^ in the electrolyte could also form a LiN_*x*_O_*y*_-based SEI and improve the performance of both Li–Cu cells and Li–metal full cells. Wu *et al.* used metal–organic frameworks (MOF-808) as nanocapsules to load LiNO_3_, and used the MOF-808/LiNO_3_ composite as an additive for LMBs (Fig. 9b).^97^ The MOF-808 has an internal diameter of 18.4 Å and a pore window of 14 Å, which can efficiently encapsulate and diffuse LiNO_3_. During battery cycling, the LiNO_3_ will be released to react with Li and form a nitrided-rich SEI. Xie *et al.* introduced nitrofullerene (nitro-C_60_) as a bifunctional electrolyte additive to smooth the Li surface.^98^ The nitro-C_60_ in the electrolyte was designed to gather on the protuberances of the Li metal electrode and decompose to NO_2_^−^ and insoluble C_60_. After that, NO_2_^−^ further reacted with Li metal and formed a compact and stable Li_3_N/LiN_*x*_O_*y*_ protective layer. The C_60_ was anchored on the uneven grooves of the Li surface and resulted in a homogeneous distribution of Li^+^ (Fig. 9c). Similarly, a paradigmatic N-rich polyether, nitrocellulose (NC), was used as an electrolyte additive to stabilize the Li metal electrode (Fig. 9d).^99^ The NC additive has low LUMO energy so that it reacts with Li to form an endogenous symbiotic Li_3_N/cellulose double SEI. However, the Li plating/stripping CE only reached ∼92%, even though the base electrolyte used ethers as the solvents.

The use of these N-containing additives also introduces extra cations and organic components into the electrolyte. Their influence on the SEI composition and the performance of LMBs has not been clearly revealed, however. In addition, as shown in Table 2, the CE for Li plating/stripping in most of these electrolytes is lower than 98%, suggesting that they are not promising for practical applications at the current stage. Also, the stability of these N-containing additives has not been studied.

**Table 2:** Summary of electrolyte engineering for constructing nitrided interfaces on Li metal electrodes

Electrolyte	N-Containing precursor	N-Containing SEI components	Current density (mA cm^−2^)	Capacity (mA h cm^−2^)	Lifespan (cycles)	Coulombic efficiency (%)	Ref.
0.38 M LiTFSI + 0.31 M LiNO_3_ + 0.23 M Li_2_S_6_ in DOL	LiNO_3_	Li_3_N and LiN_*x*_O_*y*_	N/A	N/A	N/A	N/A	87
0.5 M LiCF_3_SO_3_ + 0.4 M LiNO_3_	LiNO_3_	Li_3_N	N/A	N/A	100	90	84
0.1 M LiNO_3_ + 0.1 M Li_2_S_6_ in DOL/DME	LiNO_3_	Li_3_N and LiN_*x*_O_*y*_	N/A	N/A	N/A	N/A	85
0.5 M LiNO_3_ in DOL/DME	LiNO_3_	Li_3_N, LiN_*x*_O_*y*_ and Li_2_N_2_O_2_	N/A	N/A	N/A	N/A	110
1 M LiTFSI in DOL/DME + 0.18 M Li_2_S_8_ + 5 wt% LiNO_3_	LiNO_3_	N–S groups	2	1	400	99.1	111
2.3 M LiFSI in DME + 20 mM CuF_2_ and 20 mM LiNO_3_	LiNO_3_	Li_3_N and LiN_*x*_O_*y*_	1	1	500	99.5	112
1 M LiPF_6_ in EC/DMC with 2 v% VC + 0.1 M LiNO_3_	LiNO_3_	Li_3_N	N/A	N/A	100	∼98	90
1 M LiPF_6_ in EC/DEC with 0.2 wt% CuF_2_ and 1 wt% LiNO_3_	LiNO_3_	Li_3_N	0.5	0.5	20	98.1	91
1 M LiPF_6_ in FEC/DMC/DME with 1.1 wt% LiNO_3_	LiNO_3_	Li_3_N and LiN_*x*_O_*y*_	N/A	N/A	N/A	N/A	103
1 M LiTFSI in TEP/VC + 5% LiNO_3_	LiNO_3_	Li_3_N and N–S groups	N/A	N/A	N/A	97.3	95
1 M LiDFOB in TEP/EC with 0.2 M LiNO_3_	LiNO_3_	Li_3_N	N/A	N/A	100	∼95	113
1 M LiPF_6_ in FEC/EMC with 1 wt% TPFPB and 3 wt% LiNO_3_	LiNO_3_	Li_3_N	1	1	300	98.5	105
1 M LiPF_6_ in FEC/EMC with 0.5 wt% Sn(OTf)_2_ and 5 wt% LiNO_3_	LiNO_3_	LiN_*x*_O_*y*_	1	3	150	98.14	35
1 M LiPF_6_ EC/DEC with 10 mM In(OTf)_3_ and 0.5 M LiNO_3_	LiNO_3_	Li_3_N and LiN_*x*_O_*y*_	1	4	100	98.2	92
1 M LiFSI in FEC/GBL with 0.3 M LiNO_3_	LiNO_3_	Li_3_N and N–S groups	0.5	1	200	98.8	89
3.25 M LiTFSI in SL with 0.1 M LiNO_3_	LiNO_3_	Li_3_N and N–S groups	0.5	1	100	98.5	93
0.8 M LiPF_6_ FEC/DMC with a 5 wt% additive of 4 M LiNO_3_/DMSO	LiNO_3_	LiN_*x*_O_*y*_	1	1	100	99.42	94
1 M LiPF_6_ EC/DEC with a 50 mg mL^−1^ LiNO_3_–MOF composite	LiNO_3_-MOF	Li_3_N and LiN_*x*_O_*y*_	0.5	0.5	20	98.8	97
0.8 M LiTFSI + 0.2 M LiDFOB + 0.05 M LiPF_6_ with 0.1 M Mg(NO_3_)_2_ in EMC/FEC	Mg(NO_3_)_2_	LiN_*x*_O_*y*_ and N–S groups	2	2	100	∼94	96
1 M LiPF_6_ EC/DEC with a 5 M nitro-C_60_ derivative	Nitro-C_60_	Li_3_N and LiN_*x*_O_*y*_	0.1	0.5	150	∼92	98
1 M LiTFSI in DOL/DME with 1 wt% LiNO_3_ and ∼10 wt% TiN	Mainly TiN	TiN and N–H groups	1	1	270	97.19	114
1 M LiTFSI in DOL/DME with 2% nitrocellulose	Nitrocellulose	Li_3_N and LiN_*x*_O_*y*_	1	1	150	92	99
1 M LiTFSI in DOL/DME with 1 wt% LiNO_3_ with 0.5 mg mL^−1^ AlN	AlN	N/A	2	1	170	94.68	115
2 M LiTFSI in Py_13_TFSI/DOL/DME	Py_13_TFSI	Li_3_N and N^+^(Py_13_)	1	3	50	99.1	100
1 M LiTFSI in DOL/DME with 1 M Pyr1(4) FSI	Pyr1(4) FSI	Li_3_N and N^+^(Pyr1(4))	1	1	50	97.7	102
1 M LiTNFSI in DOL/PI_13_FASI	LiTNFSI and PI_13_FASI	Li_3_N and N^+^(PI_13_)	0.5	N/A	300	98.7	101
1 M LiTFSI in FDMA/FEC	FDMA	Li_3_N	0.5	1	100	∼99.3	107

### 4.3. N-Containing ionic liquids

**Fig. 10:**
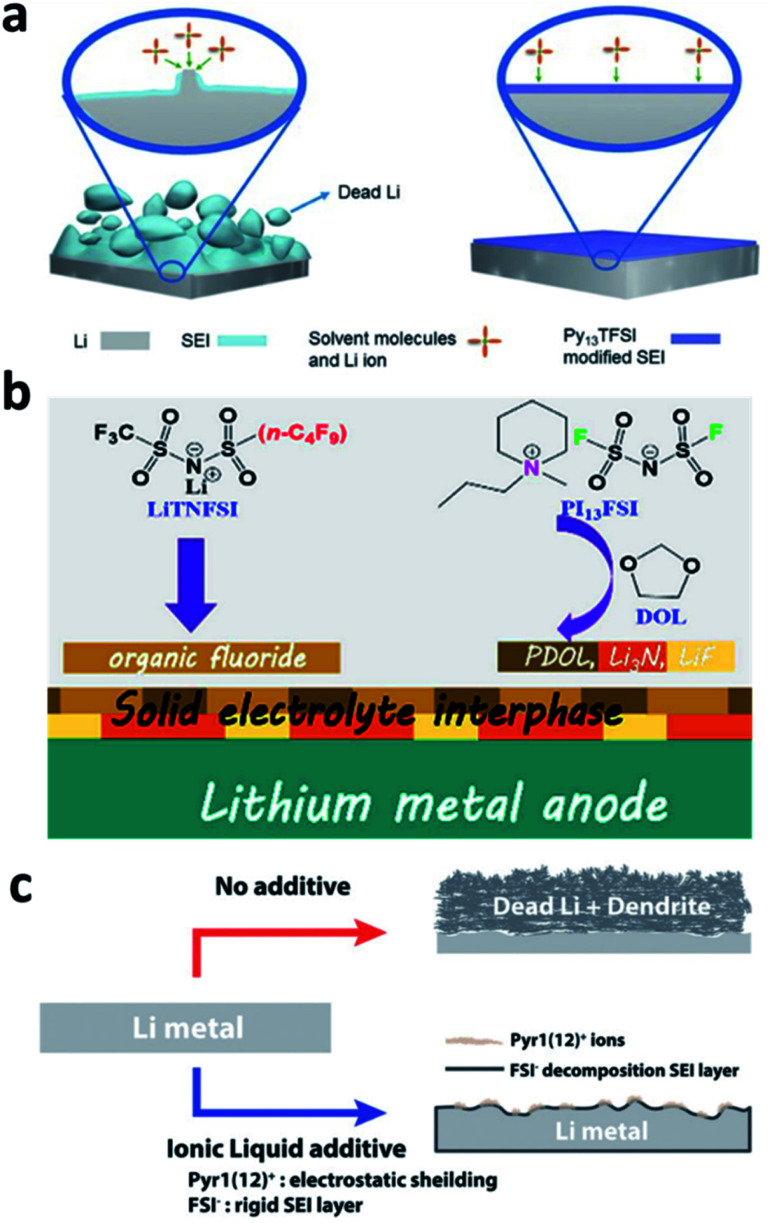
The use of an ionic liquid in the electrolyte for forming nitrided interfaces on Li metal electrodes. (a) Schematic illustration of the use of the Py_13_TFSI ionic liquid in an ether electrolyte for stabilizing the Li metal electrode;^100^ reproduced with permission. Copyright 2017, John Wiley and Sons. (b) Illustration of the co-use of the PI_13_FSI ionic liquid and LiTNFSI to construct a nitrided composite SEI on the Li metal electrode;^101^ reproduced with permission. Copyright 2018, American Chemical Society. (c) Illustration of the application of Pyr1(12)FSI to regulate the uniform deposition of Li^+^ and reduce dead Li.^102^ Reproduced with permission. Copyright 2018, John Wiley and Sons.

The decomposition of normal organic solvents on Li metal electrodes leads to the formation of organic components such as ROCOOLi or the inorganic component Li_2_CO_3_ in the SEI, both of which have ultralow Li^+^ ionic conductivity and limit the kinetics of Li plating. N-Containing ionic liquids can be used to optimize the SEI composition and generate more effective species for conducting Li^+^. Due to the high viscosity of ionic liquids, however, they are normally used in mixed solvents. For example, Guo *et al.* developed an electrolyte with a mixed solvent consisting of *N*-propyl-*N*-methylpyrrolidinium bis(trifluoromethanesulfonyl)amide (Py_13_TFSI) and normal ether solvents 1,3-dioxolane/1,2-dimethoxyethane (DOL/DME) for LMBs.^100^ The Py_13_TFSI was reduced to form N^+^(Py_13_) and N^−^(TFSI) species in the SEI, which was able to passivate the active surface of the Li electrode (Fig. 10a). In addition, more ionically conductive Li_3_N was generated on the Li surface during battery cycling. Peng *et al.* developed an electrolyte that used a mixture of *N*-propyl-*N*-methylpiperidinium bis(fluorosulfonyl)imide (PI_13_FSI) and DOL as the solvent and Li[(CF_3_SO_2_)(*n*-C_4_F_9_SO_2_)N] (LiTNFSI) as the salt.^101^ The ionic liquid and the salt decomposed on the surface of the Li metal anode to form an Li_3_N-containing SEI that was highly ionically conductive and flexible (Fig. 10b), and a CE of 98.2% was achieved in Li–Cu cells, even at a high current density of 10 mA cm^−2^. Choi *et al.* reported the use of 1-dodecyl-1-methylpyrrolidinium (Pyr1(12)^+^) bis(fluorosulfonyl)imide (FSI^−^) in ordinary electrolyte solutions.^102^ The Pyr1(12)^+^ cation with a long aliphatic chain mitigated dendrite growth *via* the synergistic effects of electrostatic shielding and lithiophobicity, and the FSI^−^ anion induced the generation of a rigid nitrided SEI (Fig. 10c).

Even the ether solvents reduce the viscosity of N-containing ionic liquid-based electrolytes, and they also limit the voltage window of the electrolyte. In addition, the high volatility and flammability of ether solvents diminish the safety advantages of ionic liquids in the electrolyte. The high price of ionic liquids is another problem that should be addressed before large-scale applications.

### 4.4. Constructing F-rich and N-rich composite interfaces

**Fig. 11:**
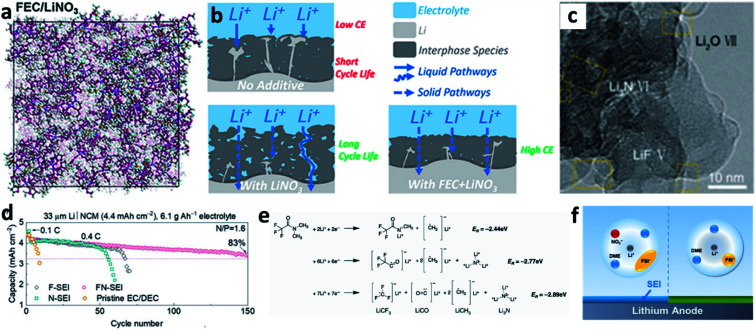
Constructing F-rich and N-rich composite interfaces for stabilizing Li metal electrodes. (a) MD results for a carbonate electrolyte with FEC as the solubilizer to improve the solvability of LiNO_3_;^103^ reproduced with permission. Copyright 2018, John Wiley and Sons. (b) Schematic illustration of the formation of a denser and more effective SEI on the Li metal electrode due to the synergistic effects of FEC and LiNO_3_ in the electrolyte;^104^ reproduced with permission. Copyright 2019, American Chemical Society. (c) Cryo-TEM image of the SEI formed in the electrolyte with tris(pentafluorophenyl)borane and LiNO_3_ as a dual additive;^105^ reproduced with permission. Copyright 2020, John Wiley and Sons. (d) Cycling performance of Li-NCM batteries with different types of SEIs;^106^ reproduced with permission. Copyright 2020, John Wiley and Sons. (e) Decomposition routes of the FDMA solvent to form Li_3_N;^107^ reproduced with permission. Copyright 2020, Springer Nature. (f) Structural illustration of the Li^+^ solvation structure of an ether electrolyte with or without NO_3_^−^.^109^ Reproduced with permission. Copyright 2019, American Chemical Society.

LiF has an ultra-high Young's modulus, so it can suppress Li dendrite growth.^49^ Although its bulk ionic conductivity is poor, it could form a compact structure and conduct Li^+^*via* grain boundaries because of the high grain boundary energy. Inorganic nitrides such as Li_3_N have much higher bulk ionic conductivity, but their particle size is larger than that of LiF, and the connections between the nitride grains are not as compact as that between LiF grains. The F-rich (LiF) and N-rich (Li_3_N and LiN_*x*_O_*y*_) composite interface is more effective for stabilizing the Li metal anode. The most common approach to introduce the LiF species on the surface of Li is using F-containing solvents. In this regard, Zhang *et al.* designed an electrolyte with a mixed carbonate ester containing fluoroethylene carbonate (FEC) as the solvent and LiNO_3_ as an additive.^103^ The FEC and LiNO_3_ in the electrolyte altered the solvation sheath of Li^+^ (Fig. 11a), and formed a uniform SEI with an abundance of LiF and LiN_*x*_O_*y*_ on the Li metal anode. Wang *et al.* pointed out that the simultaneous use of LiNO_3_ and FEC in a carbonate-based electrolyte reduced the reactivity of the electrolyte and formed a more compact SEI, thus shortening the diffusion paths of Li^+^ through the SEI and improving the CE of the resultant LMBs (Fig. 11b).^104^ Lu *et al.* dissolved 3 wt% LiNO_3_ with the aid of 1 wt% tris(pentafluorophenyl)borane as a solubilizer in FEC-based carbonate solvents and produced an nitrided and fluorinated composite SEI.^105^ The co-existence of LiF and Li_3_N in the SEI on Li was verified with a cryo-TEM (Fig. 11c). Zhang *et al.* proved that under the protection of this F- and N-rich SEI, LMBs delivered much superior electrochemical performance and a prolonged lifespan (Fig. 11d).^106^ Li *et al.* designed an electrolyte with a mixed solvent consisting of fluoro-amide (2,2,2-trifluoro-*N*,*N*-dimethylacetamide (FDMA)) and FEC.^107^ The FDMA would react with Li *via* a three-step decomposition mechanism and finally form Li_3_N (Fig. 11e), while FEC would react with Li to generate LiF on the surface of the Li metal electrode. Benefiting from the composite SEI, the plated Li was much denser and the stripping of Li was much more homogeneous. Zeng *et al.* also formulated an electrolyte with a mixture of fluorine-rich carbonate and cyclophosphonitrile as the solvent, and formed a fluoride-nitride ion-conducting interphase to suppress Li dendrite growth.^108^ Optimizing the solvation structure of the electrolyte is another approach to generate a composite SEI. For instance, Zhang *et al.* proposed that the presence of NO_3_^−^ in the electrolyte could alter the structure of the Li^+^ solvation sheath and promote the decomposition of the FSI^−^ anion, so that an SEI containing LiF and LiN_*x*_O_*y*_ was formed on the Li surface (Fig. 11f).^109^

As summarized in Table 2, the reported ether-based electrolytes normally deliver higher CE than ester-based electrolytes, because ether solvents are more stable against Li metal than ester solvents, although the low anodic decomposition voltage (<4 V) of ether-based electrolytes limits their application potential in high-voltage LMBs. In addition, although these reported electrolytes have improved the reversibility of the Li plating/stripping process, most of their CEs were still lower than 99.5%, which is not high enough for long-term and high-volumetric-energy-density LMBs. Furthermore, in most of the published results, the CE of the electrolytes was evaluated under a current density and an areal capacity lower than required for practical applications (higher than 3 mA cm^−2^ and 3 mA h cm^−2^, respectively).

To maintain reasonable viscosity and stability of the electrolyte, the amount of LiNO_3_ or other additives has been limited in the reported results. Nevertheless, these additives are continuously consumed/decomposed during battery cycling, and once the additives are depleted, the electrochemical performance of LMBs would decay rapidly. In contrast, the amounts of amides, fluoroamides, and N-containing ionic liquids are higher than that of additives when they are used as solvents in the electrolyte, and they can quickly repair the damaged SEI *via* reacting with newly exposed Li. Therefore, from the point of view of prolonging the lifespan of LMBs, the use of these N-containing solvents is more promising than the use of N-containing additives.

## 5. Substrate modification

The Li plating behaviours on the surfaces of metallic Li anodes and current collectors are quite different. The lithiophilicity of the substrate determines the over-potential for Li plating and the size of the nuclei at the initial stage of Li deposition.^33,116–119^ Nevertheless, most substrates, such as Cu, are lithiophobic.^120–122^ In addition, a practical current collector substrate is uneven with some protuberant tips on the surface, which induce a non-uniform distribution of the electric field and inhomogeneous charge distribution near the substrate, which eventually leads to the formation and growth of Li dendrites. Constructing nitrided interfaces on current collector substrates is important to improve their lithiophilicity and adjust the local electric field, thus regulating the uniform deposition of Li^+^.

### 5.1. Cu substrate

**Fig. 12:**
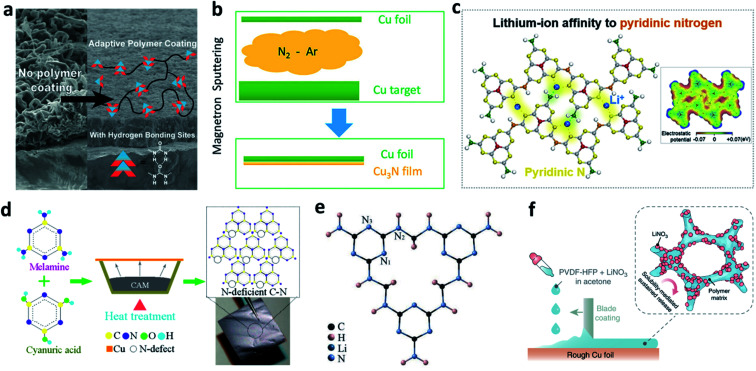
Modification of a Cu substrate with nitrided interfaces. (a) SEM images showing the effect of an adaptive polymer coated on Cu foil to suppress Li dendrite growth, and the structure of the adaptive polymer layer;^123^ reproduced with permission. Copyright 2016, American Chemical Society. (b) Schematic illustration of the preparation of a Cu_3_N layer on Cu foil by using the reaction of Cu and N_2_ gas;^124^ reproduced with permission. Copyright 2018, American Chemical Society. (c) Illustration of the pyridinic nitrogen in g-C_3_N_4_, which serves as a Li^+^ affinity centre;^125^ reproduced with permission. Copyright 2020, Elsevier. (d) Preparation of a defect-rich C–N polymer on Cu foil;^126^ reproduced with permission. Copyright 2020, American Chemical Society. (e) Structure of the poly-melamine-formaldehyde framework;^127^ reproduced with permission. Copyright 2018, John Wiley and Sons. (f) Schematic illustration of blade coating of a PVDF-HFP + LiNO_3_ layer on rough Cu foil.^128^ Reproduced with permission. Copyright 2018, Springer Nature.

Cu is the most popular substrate/current collector for negative electrodes in LMBs or anode-free batteries. Nitrided interfaces are able to guide Li^+^ homogeneous plating and improve the Li plating/stripping reversibility on a Cu substrate. Cui *et al.* synthesized an adaptive polymer with abundant N–H hydrogen bonding sites and applied it for Cu foil modification, where they achieved a much more uniform morphology of plated Li (Fig. 12a).^123^ Li *et al.* used a reactive sputtering method to develop a Cu_3_N layer on Cu foil (Fig. 12b).^124^ The Cu_3_N layer was believed to promote uniform surface electronic conductivity of the Cu foil, and it could further react with the deposited Li to form Li_3_N after the first plating process. The modified layer improved the CE in Li–Cu cells and the cycling stability of anode-free LiFePO_4_ (LFP)‖Cu cells.

In addition to its function in protecting the Li metal electrode, as discussed above, g-C_3_N_4_ can also regulate Li^+^ deposition behaviour on Cu foil. Song *et al.* reported that the pyridinic nitrogen of g-C_3_N_4_ can serve as a Li^+^ affinity centre and help to improve the lithiophobicity of Cu foil (Fig. 12c).^125^ The g-C_3_N_4_ layer can also facilitate Li^+^ conduction at the SEI through a site-to-site hopping mechanism. In addition, defect engineering of a C–N polymer was proposed to construct an N-deficient ultrathin layer (27 nm) on Cu foil *via* reactive thermal evaporation (Fig. 12d).^126^ The lithiophilicity of the defective C–N layer triggered a space charge effect in the SEI and enhanced its charge-transfer capability, leading to a lower nucleation over-potential. In addition, a three-dimensional (3D) porous poly-melamine-formaldehyde (PMF) framework was developed to modify Cu foil and prepare a PMF/Li composite anode. The amine and triazine groups in the PMF can homogenize Li^+^ concentration near the Cu surface and regulate the uniform deposition of Li (Fig. 12e).^127^

Decorating LiNO_3_ on Cu foil is also effective for forming nitrided interfaces, but the main problem is that LiNO_3_ consists of inorganic particles so that it cannot closely adhere to the Cu foil. To solve this issue, with the aid of a polymeric matrix of poly(vinylidene fluoride-*co*-hexafluoropropylene) (PVDF-HFP), Cui *et al.* coated a thin layer of LiNO_3_ on the surface of rough Cu.^128^ In this design, NO_3_^−^ can be continuously released from the layer into the carbonate-based electrolyte during the Li plating process to maintain an appreciable local NO_3_^−^ concentration at the anode surface (Fig. 12f). In addition, Xie *et al.* immersed commercially available Cu foam into LiNO_3_ aqueous solution to load LiNO_3_ particles into the pores and inner surface of the Cu foam.^129^ When operating in a carbonate-based electrolyte, the LiNO_3_ was reduced and formed an N-rich SEI on the outer and inner surfaces of the Cu foam. The authors believe that this facile method can be applied in large-scale production.

Apart from these advances, polyacrylonitrile (PAN) or PAN-based materials were used as interfacial functional layers to guide the uniform deposition of Li^+^ ions.^130,131^ AlN interlayers, which simultaneously possessed high Li affinity and an insulating nature, were also used as a surface stabilizer for Li metal anodes.^132^ Metal–organic framework (MOF) or MOF-derived materials could increase the affinity of Li^+^ to the Cu substrate, so they were also reported to suppress Li dendrite growth.^121^

### 5.2. Other substrates

**Fig. 13:**
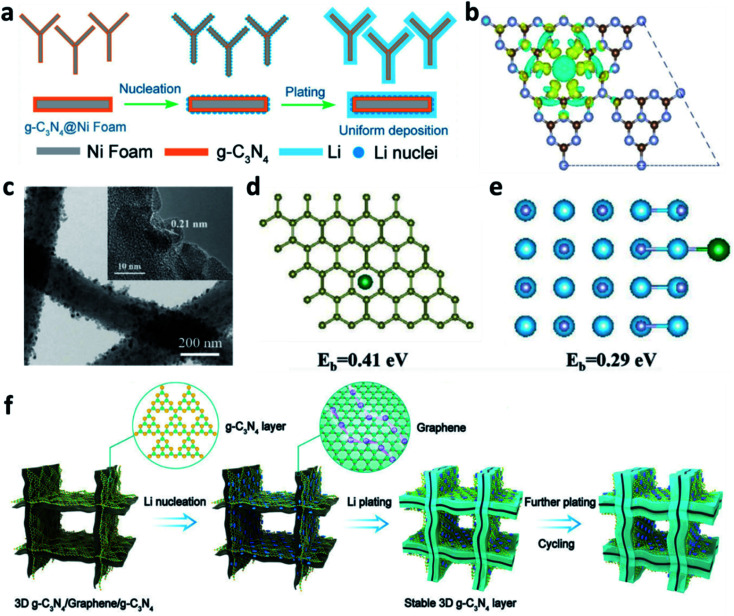
(a) Schematic illustration of the preparation of a g-C_3_N_4_ layer on Ni foam and its effects towards regulating Li^+^ uniform deposition; (b) charge distribution of the g-C_3_N_4_ layer revealing that N atoms have higher electron density and can absorb positively charged Li^+^;^134^ reproduced with permission. Copyright 2019, John Wiley and Sons. (c) TEM images of the TiN-decorated 3D carbon fibre; (d) optimized geometries for calculating the binding energy of a Li atom adsorbed on TiN, and (e) the corresponding charge density difference;^136^ reproduced with permission. Copyright 2019, John Wiley and Sons. (f) Schematic illustration of the Li deposition on the 3D g-C_3_N_4_/G/g-C_3_N_4_ electrode.^137^ Reproduced with permission. Copyright 2021, John Wiley and Sons.

3D nickel (Ni) foam can also be used as a substrate to accommodate Li. Similar to Cu, the lithiophobic and uneven surface of Ni leads to uniform deposition of Li. Compared with two-dimensional (2D) planar Cu, the 3D porous structure of Ni foam offers space to alleviate the volume changes of Li during plating/stripping processes, so it could accommodate high capacity Li plating. To improve the surface lithiophobicity of Ni, Nan *et al.* decorated cobalt nitride (Co_3_N) nanobrushes on Ni foam.^133^ The Co_3_N enabled a low over-potential for nucleation, leading to homogeneous plating of dendrite-free Li. Yang *et al.* used experimental results and theoretical simulations to prove that a micro-electric field can be formed by the tri-*s*-triazine units of g-C_3_N_4_ that were used to modify Ni foam, which induced numerous Li nuclei during the initial plating and guided the uniform growth of Li on the substrate (Fig. 13a and b).^134^ Sun *et al.* decorated Ni_*x*_N on the surface of Ni foam, which improved the specific surface area to reduce the local current density and promoted the uniform plating of Li by the formation of Li_3_N.^135^

3D carbon is also a good scaffold to host Li. The modification achieved by nitrided materials can help to increase the affinity of Li^+^ to the carbon matrix and thus decrease the over-potential for Li plating. For example, Li *et al.* prepared TiN-decorated 3D carbon fibres as a scaffold for Li metal anodes (Fig. 13c).^136^ The TiN sheath on the carbon fibre could absorb Li^+^ and reduce the diffusion energy barrier, thus providing uniform nucleation sites for Li and suppressing the formation of Li dendrites (Fig. 13d and e). Gong *et al.* decorated g-C_3_N_4_ on 3D graphene to develop a g-C_3_N_4_/graphene/g-C_3_N_4_ architecture, which can be used as an electrode to accommodate the Li metal anode (Fig. 13f).^137^ The sandwiched structure can guide uniform Li plating/stripping in the van der Waals gap between the graphene and the g-C_3_N_4_. The g-C_3_N_4_ can be regarded as an artificial SEI to prevent Li deposition on its surface and prevent the direct contact of the electrolyte with the Li metal because of its isolating nature. Other polar nitrides, such as AlN and Mg_3_N_2_, were also reported to modify 3D carbon hosts to regulate the Li plating behaviour and suppress Li dendrite growth.^115,138,139^

In a brief summary, nitrided interface modification has improved the lithiophilicity and adjusted the local charge distribution at the surface of substrates/current collectors. As summarized in Table 3, with the functionalization of nitrided interfaces, the Li plating/stripping efficiency on Cu substrates or other substrates has been effectively enhanced to 96–99%. Nevertheless, for real LMBs, especially for anode-free LMBs (without excess Li), the CE should reach a level of 99.9%. This means that the effects of nitrided interfaces need to be further improved. Furthermore, most of the reported results were obtained in ether-based electrolytes, which can undoubtedly improve the CE. As discussed above, evaluating the electrochemical performance with a high-voltage ester-based electrolyte is more practically significant. Besides, most of the reported results did not mention anode-free battery testing, while one of the most important aims of modifying substrates/current collectors is to build anode-free batteries.

**Table 3:** Summary of the substrate modification with nitrided interfaces for stabilizing Li metal electrodes

Substrate	Modification	Electrolyte	Current density (mA cm^−2^)	Capacity (mA h cm^−2^)	Lifespan (cycles)	Coulombic efficiency (%)	Ref.
2D Cu	Adaptive polymer	1 M LiTFSI in DOL/DME with 1 wt% LiNO_3_	1	1	180	97	123
2D Cu	Cu_3_N	1 M LiPF_6_ in EC/DMC	0.5	1	130	∼90	124
2D Cu	SBR + Cu_3_N	1 M LiPF_6_ EC/DEC	1	1	100	97.4	76
2D Cu	g-C_3_N_4_	1 M LiTFSI in DOL/DME	1	1	350	∼99	125
2D Cu	g-C_3_N_4_	1 M LiTFSI in DOL/DME with 0.2 M LiNO_3_	3	1	450	∼96	126
2D Cu	Polyacrylonitrile	1 M LiTFSI in DOL/DME with 2 wt% LiNO_3_	0.5	1	250	97.4	131
2D Cu	Aluminum nitride	1 M LiPF_6_ in EC/DEC with 5 v% FEC	0.5	1	125	∼97	132
2D Cu	Metal–organic framework	1 M LiTFSI in DOL/DME with 1 wt% LiNO_3_	1	1	300	99.1	121
2D Cu	MOF comprising bipyridinic nitrogen linker	1 M LiTFSI in DOL/DME with 2 wt% LiNO_3_	1	1	600	∼96	114
2D Cu	Carbon@PVDF@LiNO_3_	1 M LiPF_6_ in EC/DEC with 5 wt% VC	1	1	200	97.9	140
2D Cu	Polyethylene terephthalate	1 M LiTFSI in DOL/DME with 2 wt% LiNO_3_	1	1	100	98	120
2D Cu	Polyacrylonitrile/polyimide	1 M LiTFSI in DOL/DME with 2 wt% LiNO_3_	2	2	130	97.3	130
Rough Cu	PVDF-HFP + LiNO_3_	0.5 M LiPF_6_ EC/DEC	1	1	200	98.1	128
Ni foam	g-C_3_N_4_	1 M LiTFSI in DOL/DME with 1 wt% LiNO_3_	2	2	140	97	134
2D Cu	3D porous polymelamine-formaldehyde	1 M LiTFSI in DOL/DME with 2 wt% LiNO_3_	10	1	50	94.7	127
Carbon nanofiber mat	TiN	1 M LiTFSI in DOL/DME with 1 wt% LiNO_3_	2	1	250	97.5	136
Ni foam	Co_3_N_4_ nanobrush	1 M LiTFSI in DOL/DME with 2 wt% LiNO_3_	1	1	120	96.9	133
3D carbon paper	Mg_3_N_2_	1 M LiTFSI in DOL/DME with 1 wt% LiNO_3_	0.5	0.5	240	98.2	139
Ni foam	Ni_*x*_N (*x* = 3, 4)	1 M LiTFSI in DOL/DME with 1 wt% LiNO_3_	1	1	300	97	135
Cu foam	LiNO_3_	1 M LiPF_6_ in EC/DEC with 10% FEC	1	1	300	95.5	129
3D graphene	g-C_3_N_4_	1 M LiTFSI in DOL/DME with 1 wt% LiNO_3_	1	1	500	99.1	137

It is worth noting that 3D Cu, Ni foam and 3D carbon hosts are all electronically conductive, so Li^+^ from the electrolyte may accept electrons and be plated on their upper surfaces (separator side). Once this occurs, the effects of the 3D structure towards accommodating Li will be greatly weakened, while the volumetric energy density of the Li anode will be sacrificed. As modified nitrides normally have poor electronic conductivity, they could decrease the surface electronic conductivity of the host and thus force Li^+^ diffusion into the pores and lead to Li deposition on the inner surface of the 3D scaffolds.

## 6. Separator functionalization

**Fig. 14:**
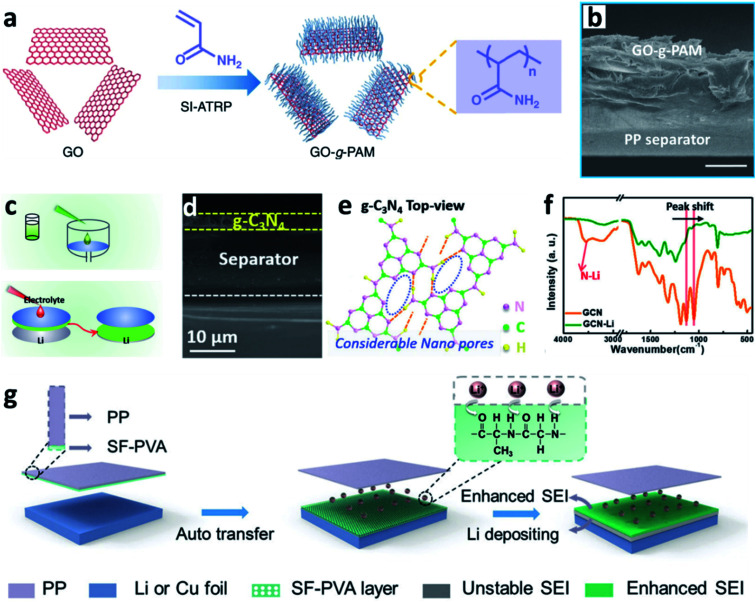
Separator functionalization for constructing nitrided interfaces on Li metal electrodes. (a) Schematic illustration of the synthesis of GO-*g*-PAM molecular brushes; (b) cross-sectional SEM images of the GO-*g*-PAM@PP separator; scale bar: 20 μm;^141^ reproduced with permission. Copyright 2019, Springer Nature. (c) Preparation of a g-C_3_N_4_ layer on the PP separator; (d) SEM image of the g-C_3_N_4_ layer supported on the PP separator; (e) Fourier transform infrared (FT-IR) spectra showing the formation of transient Li–N bonds in the g-C_3_N_4_ layer after it is immersed in the electrolyte; (f) schematic illustration of the process of Li^+^ adsorption and the formation of Li–N bonds in the g-C_3_N_4_ layer;^142^ reproduced with permission. Copyright 2019, John Wiley and Sons. (g) Schematic illustration of the modification of the SF–PVA layer on a Li metal electrode and its function in forming an enhanced SEI.^143^ Reproduced with permission. Copyright 2021, John Wiley and Sons.

Separators play a key role in all batteries. In LIBs and LMBs, the separator is a porous membrane placed between the positive electrode and negative electrode, which is permeable to ionic flow but prevents electric contact between the electrodes.^144^ Previous results proved that coating a polypropylene (PP)-based separator with BN nanosheets was useful for suppressing Li dendrite growth and prolonging the lifespan of LMBs.^145^ The separator can also be used to support N-containing materials to induce nitriding of the interface on the Li metal anode. Wang *et al.* creatively immersed a glass fibre separator in a LiNO_3_ solution to impregnate the separator with sub-micrometer-scale particles of LiNO_3_.^88^ During battery cycling, the LiNO_3_ was released into the carbonate-based electrolyte and reacted with Li to form a nitrided protective layer, which could suppress the formation of Li dendrites and “dead” Li. Li *et al.* proposed a similar concept for sustainably releasing NO_3_^−^ in a carbonate electrolyte by intercalating superfluous LiNO_3_ particles between bi-layer polypropylene membranes (PP/LiNO_3_/PP).^146^ Wu *et al.* developed a composite separator coated with polyacrylamide-grafted graphene oxide molecular brushes (GO-*g*-PAM) (Fig. 14a and b).^141^ The polyacrylamide chains contained abundant N–H and C=O groups and thus enabled a molecular-level homogeneous and fast Li^+^ flux on the surface of Li. Besides, a layer of g-C_3_N_4_ on commercially available PP separators was prepared (Fig. 14d and e), and the g-C_3_N_4_ on the PP film was grafted to the Li metal surface after cell assembly.^142^ It was proposed that the g-C_3_N_4_ can form transient Li–N bonds at the electrode/electrolyte interface to effectively stabilize the Li^+^ flux and thus enable smooth Li deposition at high current densities and capacities (Fig. 14f and g). Besides, Huang *et al.* modified a hybrid layer of silk fibroin and polyvinyl alcohol (SF–PVA) on a PP separator *via* a freeze drying method. The SF–PVA layer will auto-transferred from the PP separator to the Li surface (Fig. 14h).^143^ The N–H and C=O groups in SF are able to regulate the Li^+^ flux distribution, and the SF–PVA layer can form a Li_3_N rich SEI. Therefore, uniform Li nuclei deposition was achieved.

The separator functionalization strategies can remarkably improve the lifespan and the electrochemical performance of Li metal electrodes, even under high current density and high capacity conditions. These strategies are also convenient for large-scale production. Unfortunately, the introduction of N-containing materials increases the overall thickness of the separator, which will certainly sacrifice the volumetric energy density of LMBs.

## 7. Prospects and outlook

Nitrided interfaces have effectively stabilized Li metal electrodes and improved their electrochemical performance as well as lifespan of LMBs. Despite this success, many critical issues and challenges remain to be carefully considered in the future. They are summarized below.

### 7.1. Investigating how nitrided interfaces affect the stripping process

The majority of research has only focused on the plating process, while the stripping process, deciding the utilization of deposited Li, was rarely noticed. There is no doubt that a uniform stripping step will lead to less pulverization and depletion of active Li during each cycle, which is a significant factor in promoting the lifespan and electrochemical performance of LMBs. The course of Li dissolution with and without nitrided interfaces during the stripping step deserves to be carefully studied.

### 7.2. Understanding how nitrided interfaces affect the components and microstructure of the final SEI layer

The introduction of nitrogenous additives will definitely influence the solvent structure of the electrolyte, changing the final decomposition products, while for nitrided interfaces fabricated *ex situ*, their existence also alters the decomposition of the electrolyte. In short, the final SEI layer is composed of nitrogenous compounds and other components, and these products and their distributions have a direct correlation with the Li ion transport. If we only target nitrogenous compounds, we cannot achieve a comprehensive outlook with respect to the interfaces. A better knowledge of the interaction between nitrogenous compounds and other components will help to understand the differences in performance among the various nitrided interfaces.

### 7.3. Developing high-voltage N-containing electrolytes

The application of LiNO_3_ and other N-containing additives is mainly confined to low-voltage electrolytes due to their low solubility in most high-voltage non-aqueous solvents. Increasing their solubility in high-voltage electrolytes is of great importance.

### 7.4. Controlling the thickness of nitrided interfaces

The thickness of reported nitrided or nitride-based composite interfaces varies from a few nanometres to more than 20 μm. To avoid the sacrifice of the volumetric-energy-density of the Li metal battery, the thickness of the modification layers should be much thinner than that of the Li foil, especially considering that the Li foil used in the practical batteries is only 50 μm in thickness.

### 7.5. Improving the lifespan of the nitrided artificial SEI

The reported nitrided artificial SEI layers do exhibit positive effects towards suppressing Li dendrite growth and passivating the active Li surface, but their stability is far from satisfactory. They may be destroyed by the interfacial stress resulting from the huge volume changes of the Li metal electrode, so the real effects of nitrided artificial SEI layers would be sacrificed. Developing flexible and robust nitrided artificial SEI layers with longer lifespans is critical to promoting their practical effects.

### 7.6. Evaluating the effects of nitrided interfaces under practical conditions

According to the reported designs, most of the electrochemical performance was tested under mild conditions different from the real application situation. To make it more objective, the electrolyte and Li metal should be well quantified, while the cathode capacity should reach the commercial scale. In consideration of the mass energy density and the cost, the amount of electrolyte should be limited to less than 10 μL mA h^−1^ (lean electrolyte). Meanwhile, the areal capacity of the cathode should be higher than 3 mA h cm^−2^, and the thickness of the Li metal anode should be less than 50 μm (∼10 mA h cm^−2^), with the capacity ratio of the negative electrode (Li) to the positive electrode (n/p ratio) lower than 3. In addition, the current density and areal capacity in coulombic efficiency and symmetric cell tests should be increased to higher than 3 mA cm^−2^ and 3 mA h cm^−2^, respectively.

## Data availability

There is no origional experimental or computational data associated with this article, as it is a *Perspective*.

## Author contributions

Z. Wang and Y. Wang wrote the manuscript. Z. Guo supervised this project. All the authors discussed and polished the manuscript.

## Conflicts of interest

There are no conflicts to declare.
